# Descending Thoracic Aortic Aneurysm due to *Brucella melitensis*

**DOI:** 10.1155/2019/4939452

**Published:** 2019-09-25

**Authors:** Mohammed Alsheef, Saud Alsaleh, Nahar Alanezi, Nizar Bakhsh, Rana AlDosary, Lina AlSharif, Arshad Mian, Azeem Ahsan, Isamme AlFayyad

**Affiliations:** ^1^Department of Internal Medicine, King Fahad Medical City, P.O. Box 59046, Riyadh 11525, Saudi Arabia; ^2^King Fahad Medical City, P.O. Box 604, Riyadh 11321, Saudi Arabia; ^3^Infectious Disease, King Fahad Medical City, P.O. Box 59046, Riyadh 11525, Saudi Arabia; ^4^Research Center, King Fahad Medical City, P.O. Box 59046, Riyadh 11525, Saudi Arabia

## Abstract

Brucellosis is an endemic infection mainly in the Middle East and the Mediterranean region that can involve any system. However, cardiovascular involvement commonly seen as endocarditis is a rare occurrence, but it is one of the main causes of mortality and morbidity. *Brucella* mycotic aneurysms are extremely rare and carry a higher morbidity and mortality. Here, we present a case of *Brucella* mycotic aneurysms in the descending aorta complicated by an aortoesophageal fistula in a 52-year-old diabetic man. The diagnosis was made by thoracic CT angiogram showing a saccular aneurysm arising from the descending aorta and two positive cultures of *Brucella melitensis*. Transthoracic and transesophageal echocardiograms ruled out infective endocarditis. Aortoesophageal fistula complicating a *Brucella* mycotic aneurysm in the absence of evidence of endocarditis has not yet been reported in the literature.

## 1. Introduction

Brucellosis is a zoonotic disease found more commonly in endemic areas (Middle East and Mediterranean regions) that can affect virtually any system in the human body. However, cardiovascular complications are a rare occurrence, infective endocarditis (IE) being the most common presentation. A meta-analysis was done in Turkey, evaluating 4204 brucellosis patients reported IE in 1.2% of cases [1]. Furthermore, aortic mycotic aneurysm due to *Brucella melitensis* is a much rarer condition, a review of literature identified cases [2]. To our knowledge, an aortoesophageal fistula complicating a *Brucella* mycotic aneurysm has not yet been reported in the literature.

## 2. Case Description

**Figure 1:**
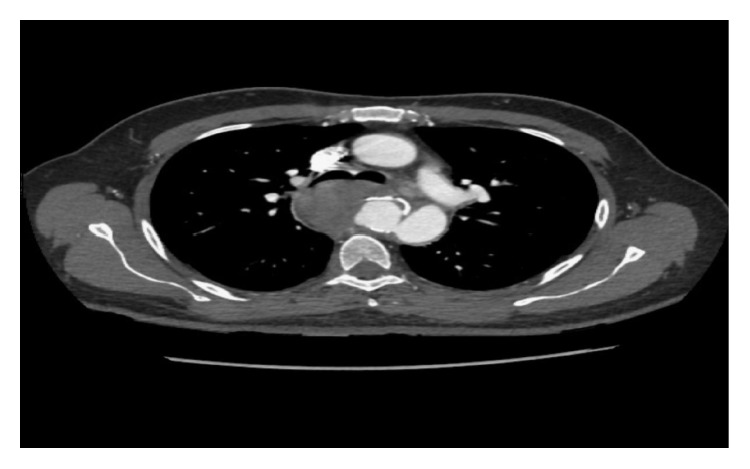
CT chest shows large saccular aneurysm in the proximal part of the descending aorta.

**Figure 2:**
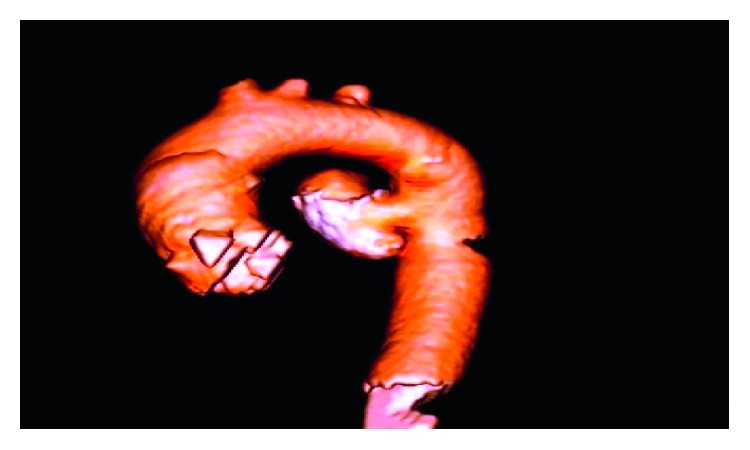
CT 3D shows aneurysm formation in descending aorta.

**Figure 3:**
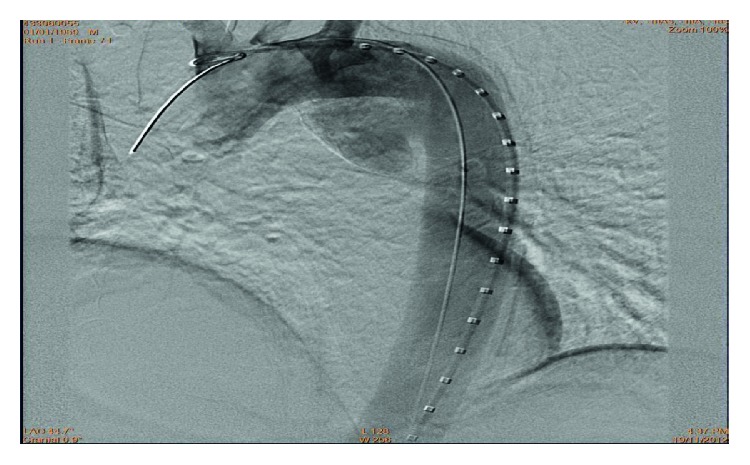
Angiogram shows endovascular repair.

**Figure 4:**
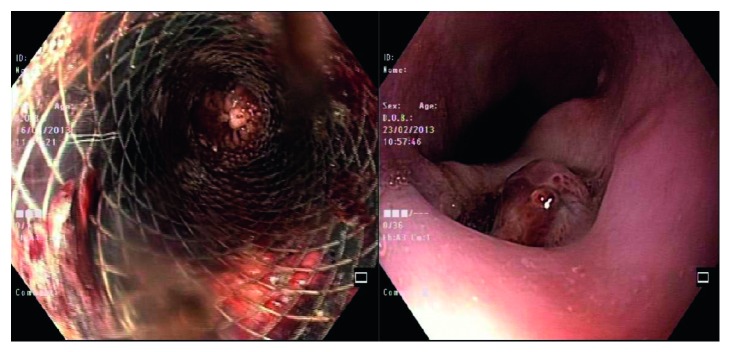
Endoscopy shows aortoesophageal fistula and stent repair.

A 52-year-old male, with long-standing type 2 diabetes mellitus, presented to our emergency department (ED) with a history of central, dull chest pain radiating to the back. He complained of intermittent high-grade fever, chills, rigors, sweating, unintentional weight loss, and dysphagia for three months. History was positive for raw milk ingestion and animal contact. Vitals revealed a temperature of 39.1°C; physical exam was unremarkable. Admission laboratory data showed white blood cells 10 (10*e*9/L), hemoglobin (16.1 mg/dl), platelet count 350 (10*e*9/L), C-reactive protein 28.5 (mg/dl), and normal electrolytes and coagulation profile. Thoracic CT angiogram demonstrated a saccular aneurysm arising from the descending aorta, distal to origin of the left subclavian artery, with perianeurysmal hematoma compressing the carina, main bronchi, and esophagus (Figure 1). Two sets of blood cultures were positive for *Brucella melitensis*. Transthoracic and transesophageal echocardiograms ruled out IE. *Brucella* mycotic aneurysm was diagnosed; appropriate intravenous and oral antibiotics (rifampin and doxycycline) were initiated. During hospitalization, the patient developed massive hematemesis necessitating transfusion of six units packed red blood cells. Angiography exhibited an aortoesophageal fistula (Figure 2), and urgent endovascular repair of the aneurysm was done (Figure 3). Subsequent angiography demonstrated satisfactory stent position with no endovascular leak. The patient received intravenous gentamicin for sixteen days along with rifampin and doxycycline. Upon discharge, rifampin and doxycycline were continued in an oral form for a total of six months. He returned to ED with recurrent hematemesis. CT and conventional angiography revealed no endovascular leak. The patient was discharged home. He again revisited with massive hematemesis with a hemoglobin drop, but no endovascular leak was found on imaging. Blood transfusions were given. Vascular surgeons decided to re-stent, as the thoracic surgeon deemed patient unsuitable for invasive surgical intervention for the aortoesophageal fistula. Esophageal stenting was done (Figure 4), but the patient could not tolerate oral intake, requiring stent removal. The following day the patient became unstable, arrested, and died.

## 3. Discussion

Brucellosis is a common zoonotic disease worldwide, *Brucella melitensis* being the most frequent organism [3, 4]. Transmission occurs after ingestion of unpasteurized dairy products and contaminated meat [3]. Brucellosis can present with a variety of clinical manifestations and virtually affecting any system [5]. Aortic brucellosis has an insidious and nonspecific presentation, requiring a high index of suspicion. Any acute pain, whether chest or abdominal with accompanying fever should raise suspicion of aortic involvement. However, there is very scarce information on the presentation patterns, appropriate investigation, and treatment of aortic brucellosis.

Furthermore, mycotic aneurysmal involvement happens due to either direct extension of an adjacent soft tissue infection or embolization of an infectious source. It is thought that in cases of embolization, the emboli reach the adventitia through the vasa vasorum and the inflammation disrupts both the muscularis and adventitia, resulting in blood vessel wall weakness [6]. Human brucellosis requires prolonged periods of multiple antibiotic therapies to completely treat the infection. In cases of cardiovascular complications, treatment becomes more difficult resulting in significant morbidity and mortality [7]. Medical treatment alone has shown to increase mortality rates (33%) when compared to both surgical and medical intervention [8–11]. Most successfully managed cases were treated with the combination of both medical and surgical intervention [8, 12, 13]. However, treatment of *Brucella* aortitis and its complications has not been well-established due to the low number of reported cases.

## 4. Conclusion

Mycotic aneurysm of the aorta due to *Brucella* is a rare and dangerous complication that needs a high index of suspicion to diagnose. When complicated with an aortoesophageal fistula, it becomes a fatal disease that requires aggressive medical and surgical intervention.
